# Distinct expression patterns for type II topoisomerases IIA and IIB in the early foetal human telencephalon

**DOI:** 10.1111/joa.12416

**Published:** 2015-11-27

**Authors:** Lauren F. Harkin, Dianne Gerrelli, Diana C. Gold Diaz, Chloe Santos, Ayman Alzu'bi, Caroline A. Austin, Gavin J. Clowry

**Affiliations:** ^1^ Institute of NeuroscienceNewcastle UniversityNewcastle upon TyneUK; ^2^ Institute of Genetic MedicineNewcastle UniversityNewcastle upon TyneUK; ^3^ HDBR ResourceUCL Institute of Child HealthLondonUK; ^4^ Institute for Cell and Molecular BiosciencesNewcastle UniversityNewcastle upon TyneUK

**Keywords:** autism susceptibility genes, cortical development, DNA replication, ganglionic eminences, RNA transcription

## Abstract

TOP2A and TOP2B are type II topoisomerase enzymes that have important but distinct roles in DNA replication and RNA transcription. Recently, TOP2B has been implicated in the transcription of long genes in particular that play crucial roles in neural development and are susceptible to mutations contributing to neurodevelopmental conditions such as autism and schizophrenia. This study maps their expression in the early foetal human telencephalon between 9 and 12 post‐conceptional weeks. TOP2A immunoreactivity was restricted to cell nuclei of the proliferative layers of the cortex and ganglionic eminences (GE), including the ventricular zone and subventricular zone (SVZ) closely matching expression of the proliferation marker KI67. Comparison with sections immunolabelled for NKX2.1, a medial GE (MGE) marker, and PAX6, a cortical progenitor cell and lateral GE (LGE) marker, revealed that TOP2A‐expressing cells were more abundant in MGE than the LGE. In the cortex, TOP2B is expressed in cell nuclei in both proliferative (SVZ) and post‐mitotic compartments (intermediate zone and cortical plate) as revealed by comparison with immunostaining for PAX6 and the post‐mitotic neuron marker TBR1. However, co‐expression with KI67 was rare. In the GE, TOP2B was also expressed by proliferative and post‐mitotic compartments. *In situ* hybridisation studies confirmed these patterns of expression, except that *TOP2A* mRNA is restricted to cells in the G2/M phase of division. Thus, during early development, TOP2A is likely to have a role in cell proliferation, whereas TOP2B is expressed in post‐mitotic cells and may be important in controlling expression of long genes even at this early stage.

## Introduction

The helical structure and supercoiling of DNA is essential for nuclear packaging; however, processes such as DNA replication and transcription require complete separation and partial separation of the strands, respectively. During transcription, the partial separation of the DNA allows RNA polymerase and transcription factors to access specific gene regions, creating tension in the DNA. Topoisomerase enzymes govern the topological state of DNA in both prokaryotic and eukaryotic cells, allowing unwinding of the DNA and relieving the torsional strain created by supercoiling (Nitiss, 2009a). Cells have type I and II topoisomerase enzymes. Type I topoisomerases (TOP1 and TOP3) are able to break a single strand of DNA, allowing the intact strand to pass through it before re‐joining the broken strand, whilst type II topoisomerases (TOP2) carry out ATP‐mediated strand breakage of one or both strands. Human topoisomerases comprise distinct alpha and beta isoforms (Austin & Marsh, 1998). Topoisomerase poisons are effective anti‐cancer drugs as they prevent cell replication and induce apoptosis (Nitiss, 2009b).

In rodents, expression of Top2a and Top2b in the brain is higher during early embryogenesis compared with the later stages (Capranico et al. 1992). However, there is a surge of Top2b expression in the brain of newborn mice that is not observed for Top2a or the marker of cell proliferation thymidylate synthase. Top2a expression is most apparent in proliferating tissues whereas Top2b expression is not confined to regions of proliferation, suggesting a role for rodent Top2b outside of DNA replication (Capranico et al. 1992; Tsutsui et al. 1993; Watanabe et al. 1994). Top2b is the predominantly expressed topoisomerase enzyme in mouse neurons (Tiwari et al. 2012), and has been shown to play a role in the development and lamination of the neocortex (Yang et al. 2000; Lyu & Wang, 2003). By E16.5, Top2b protein is found in the proliferative regions of the ventricular zone (VZ) and the subventricular zone (SVZ) of the mouse neocortex as well as in the post‐mitotic regions of the cortical plate (CP), subplate (SP) and marginal zone (MZ). Homozygous mutants exhibit abnormal cortical organisation at E16.5 with the SP being absent and the VZ appearing thickened compared with wild type (WT) (Lyu & Wang, 2003). As these defects appear late in development and expression is seen in areas containing differentiated neurons, again this suggests a role for Top2b outside of DNA replication. Table 1 provides a summary of what is known about expression, localisation and function of type 2 isomerases in mouse and rat.

**Table 1:** Topoisomerase 2 nervous system expression, localisation and function in animal models

	Mouse		Rat	
	Top2a	Top2b	Top2a	Top2b
Development	Mainly expressed in proliferative tissues (Capranico et al. 1992)	Expressed in both proliferating and non‐proliferative tissues (Capranico et al. 1992)	Transcripts highly expressed in embryonic brain but undetectable 4 weeks after birth (Tsutsui et al. 1993)	Transcripts present throughout embryonic and postnatal stages (Tsutsui et al. 1993)
		An absence of Top2b affects cerebral stratification as well as causing a lack of axon innervation in skeletal muscles and spinal cord (Yang et al. 2000; Lyu & Wang, 2003)	In the developing cerebellum, Top2a expression is confined to proliferative layer (Tsutsui et al. 1993)	In the developing cerebellum, Top2b expression is seen throughout cortical region (Tsutsui et al. 1993)
		There is a shift from Top2a to Top2b expression during neuronal differentiation (Tiwari et al. 2012)	Expressed in the VZ of embryonic brains (E13–15) (Watanabe et al. 1994)	Expressed throughout the brain from E13 to P21(Watanabe et al. 1994)
Maturity	Low expression in adult brain tissue relative to other tissues (Capranico et al. 1992)	High expression in adult brain relative to other tissues (Capranico et al. 1992)		

VZ, ventricular zone.

TOP2B has been implicated in transcriptional regulation in human cell lines (Ju et al. 2006; Perillo et al. 2008; Haffner et al. 2010; McNamara et al. 2010; Thakurela et al. 2013; Isik et al. 2015), and topoisomerases are known to be expressed in the developing and adult human brain (Zandvliet et al. 1996; Plaschkes et al. 2005). Evidence suggests that topoisomerase mutations can potentially affect the expression of susceptibility genes of neurodevelopmental disorders, including autism spectrum disorder (ASD; King et al. 2013) Fragile X syndrome (Xu et al. 2013) and mental retardation (Tarsitano et al. 2014). It has been shown that TOP2B may be required for efficient transcription of a subset of long genes linked to ASD with roles in synapse formation and stabilisation (King et al. 2013). This raises the possibility that topoisomerase proteins may have developmental roles in establishing the neural circuitry essential for the proper functioning of the human brain.

This study focusses on the expression of *TOP2A* and *TOP2B* in the developing human brain as they are highly expressed in neuronal cells. For the first time, the spatial expression of TOP2A and TOP2B was looked at across the developing human telencephalon from 9 to 12 post‐conceptional weeks (PCW) using histochemical techniques. This time period covers the end of embryogenesis and the onset of the foetal stages, from the beginning of the formation of the CP but before thalamic afferents have innervated the cerebral cortex. In order to interpret these expression patterns more easily, nearby sections were immunostained for proteins that have been validated as markers for known human cell types/compartments (Bayatti et al. 2008a; Pauly et al. 2014), including PAX6 (radial glia), KI67 (dividing cells), TBR1 (early post‐mitotic cortical neurons) and NKX2.1 [progenitor cells of the medial ganglionic eminence (MGE)].

## Materials and methods

### Human tissue

Human embryonic and foetal brain sections between 8 and 12 PCW were obtained from the MRC‐Wellcome Trust Human Developmental Biology Resource (HDBR, http://www.hdbr.org). Brains were collected from terminations of pregnancy with maternal consent with the approval of local ethical committees. Age was determined by the assessment of external morphology (O'Rahilly et al. 1987; Bullen & Wilson, 1997) or by comparing foot and heel to knee length with a standard growth chart (Hern, 1984).

### Immunohistochemistry (IHC)

Brains were fixed in buffered 4% paraformaldehyde solution (PFA; Sigma Aldrich, Dorset, UK) and embedded in paraffin wax before sectioning. Deparaffinised coronal and sagittal sections collected on slides were immersed in 1.5% hydrogen peroxide : methanol solution (Sigma Aldrich) to block activity of endogenous peroxidases. Heat‐mediated antigen retrieval was carried out by treatment of sections in citrate buffer (pH 6.0) before incubating sections with 10% of the appropriate normal serum (Vector Laboratories, Peterborough, UK) in Tris‐buffered saline (TBS) for 10 min. For immunoperoxidase staining, sections were then incubated with a primary antibody (Tables 2 and 3 for source of antibodies and dilutions used) in TBS solution overnight at 4 °C. Sections were then washed and incubated for 30 min with the appropriate biotinylated secondary antibody (Vector Laboratories), washed then incubated for 30 min with Vectastain Elite ABC kit (Vector Laboratories) and developed using 3,3′‐diaminobenzidine (Vector Laboratories). Sections were dehydrated, cleared and coverslipped.

**Table 2:** Primary and secondary antibodies used in this study

Primary antibody	Species	Dilution	Supplier
KI67	Mouse	1/150	Dako, Cambridgeshire, UK, clone MIB1
NKX2.1	Mouse	1/150	Dako, Cambridgeshire, UK
PAX6	Rabbit polyclonal	1/1500	Covance, Cambridge Bioscience, UK
TBR1	Rabbit polyclonal	1/1500	Abcam, Cambridge, UK
TOP2A	Rabbit polyclonal	1/800	Prof. Caroline Austin^a^
TOP2B	Rabbit polyclonal	1/800	Prof. Caroline Austin^a^

a From research funded by Leukaemia and Lymphoma Research.

**Table 3:** Immunogen sequences detected by polyclonal rabbit antibodies raised against TOP2A and TOP2B

Gene	Immunogen sequence	Length/aa
**TOP2A**	EGSPQEDGVELEGLKQRLEKKQKREPGTKTKKQTTLAFKPIKKGKKRNPWSDSESDRSSDESNFDVPPRETEPRRAATKTKFTMDLDSDEDFSDFDEKTDDEDFVPSDASPPKTKTSPKLSNKELKPQKSVVSDLEADDVKGSVPLSSSPPATHFPDETEITNPVPKKNVTVKKTAAKSQSSTSTTGAKKRAAPKGTKRDPALNSGVSQKPDPAKTKNRRKRKPSTSDDSDSNFEKIVSKAVTSKKSKGESDDFHMDFDSAVAPRAKSVRAKKPIKYLEESDEDDLF	287
**TOP2B**	LDTAAVKVEFDEEFSGAPVEGAGEEALTPSVPINKGPKPKREKKEPGTRVRKTPTSSGKPSAKKVKKRNPWSDDESKSESDLEETEPVVIPRDSLLRRAAAERPKYTFDFSEEEDDDADDDDDDNNDLEELKVKASPITNDGEDEFVPSDGLDKDEYTFSPGKSKATPEKSLHDKKSQDFGNLFSFPSYSQKSEDDSAKFDSNEEDSASVFSPSFGLKQTDKVPSKTVAAKKGKPSSDTVPKPKRAPKQKKVVEAVNSDSDSEFGIPKKTTTPKGKGRGAKKRKASGSENEGDYNPGRKTSKTTSKKPKKTSFDQDSDVDIFPSDFPTEPPSLPRTGRARKEVKYFAESDEEEDDVDF	358

For immunofluorescent double‐labelling, a novel procedure that allows two polyclonal primary antibodies to be employed was developed from the methods of Goto et al. (2015). Sections were incubated with the first primary antibody as before, washed and incubated with ImmPRESS™ HRP IgG (Peroxidase) Polymer Detection Kit, made in horse (IF; Vector Laboratories), washed then incubated for 30 min with tyramide signal amplification (TSA™) fluorescein plus system reagent (IF; Perkin Elmer, London, UK). After washing but before application of a second primary antibody, heat‐mediated antigen retrieval was carried out by treatment of sections in citrate buffer (pH 6.0). This removes the first set of primary and secondary antibodies employed, but leaves fluorescent tyramide covalently bound to the tissue section. The method above was repeated for the detection of this second primary antibody, except that tyramide signal amplification (TSA™) rhodamine plus was used for detection. Sections were then washed, stained with 4′,6‐diamidino‐2‐phenylindole, dihydrochloride (DAPI; Thermo Fisher Scientific), washed and coverslipped with Vectashield (Vector Laboratories).

### Manufacture of *in situ* hybridisation (ISH) probes

Polymerase chain reaction (PCR) using specific primers (Sigma Aldrich; Table 4) amplified the required DNA fragment of the gene of interest using human brain foetal DNA as a template, which was run on a gel to confirm the correct size of the product. The band was gel extracted (Qiagen, Crawley, UK), and the DNA was cloned and transformed into competent cells using the pGEM^®‐^T Easy kit (Promega, Southampton, UK). Select colonies were grown in 5 mL LB‐Broth (Invitrogen, Paisley, UK) with ampicillin (overnight). Plasmids were prepared using the Miniprep kit (Qiagen) and the HiSpeed plasmid Maxiprep kit (Qiagen) before restriction digest using the Qiagen PCR purification kit. Digoxigenin‐UTP was incorporated into riboprobes during *in vitro* transcription using the DIG RNA Labelling Kit (SP6/T7; Roche, Indianapolis, USA) according to the manufacturer's instructions. Antisense and sense probes were generated using T7 and SP6 polymerase, respectively. Probe concentration was measured (Nanodrop; Thermo Scientific, Waltham, USA).

**Table 4:** Primers used to manufacture probes used in the detection of TOP2A, TOP2B and TBR1 mRNA sequences

Gene	Forward primer 5′–3′ sequence	Reverse primer 5′–3′ sequence	Product size/bp
TBR1	TAAGTTAATACGACTCACTATAGGGCGA	AATACGATTTAGGTGACACTA TAGAATAC	426
TOP2A	GCCCAAGACTGGTTTTAAAGTT	TGGAGATTTCCCAAAATGAATC	800
TOP2B	TTGGTCAGATGATGAATCCAAG	AAGACGGTTTTCCCTTTTTAGC	500

### Tissue *in situ* hybridisation (TISH)

Paraffin sections were dewaxed in two changes of histoclear and gradually hydrated in decreasing ethanol concentrations. Sections were washed twice in PBS fixed for 20 min in PFA. Sections were washed twice in PBS and then incubated with proteinase K (20 μL mL^−1^; Sigma Aldrich) for 8 min and post‐fixed in 4% PFA : PBS (Sigma) at room temperature for 5 min. After two washes in PBS, sections were treated with 0.1 m tetraethylammonium/0.25% acetic anhydride for 10 min, washed again twice in PBS. Dehydrated and air‐dried slides were covered with 300 ng DIG‐labelled riboprobe, hybridisation mix (50% formamide, 0.3 m NaCl, 20 mm Tris pH 8.5, 5 mm EDTA pH 8.0, 1 × Denharts solution, 10% dextran sulphate), tRNA (0.5 mg mL^−1^) and RNAse inhibitor (1 μL mL^−1^), coverslipped and the mixture was incubated overnight at 65 °C to allow hybridisation of probes to the tissue mRNA. Post‐hybridisation washes were performed [2 × standard sodium citrate (SSC), 65 °C; 0.2 × SSC, 65 °C; 2 × formamide wash (350 mL formamide, 70 mL 20 × SSC, 280 mL dH_2_O)], and slides were incubated for 1 h in 150 mm NaCl and 100 mm Tris–HCl, pH 7.5, containing 10% foetal calf serum followed by incubation with anti‐DIG : alkaline phosphatase and expression was visualised using NBT/BCIP (Roche), sections mounted using Vectamount (Vector Laboratories).

### Imaging

Images were taken using an Axioimager Z2 microscope equipped with an Axiocam and Axioimager Z2 4.8.1 software or scanned using the Leica SCN400 Slide Scanner. Final images for publication were produced using Adobe Photoshop CS6 software.

## Results

### TOP2A is expressed in regions of cell division

**Figure 1:**
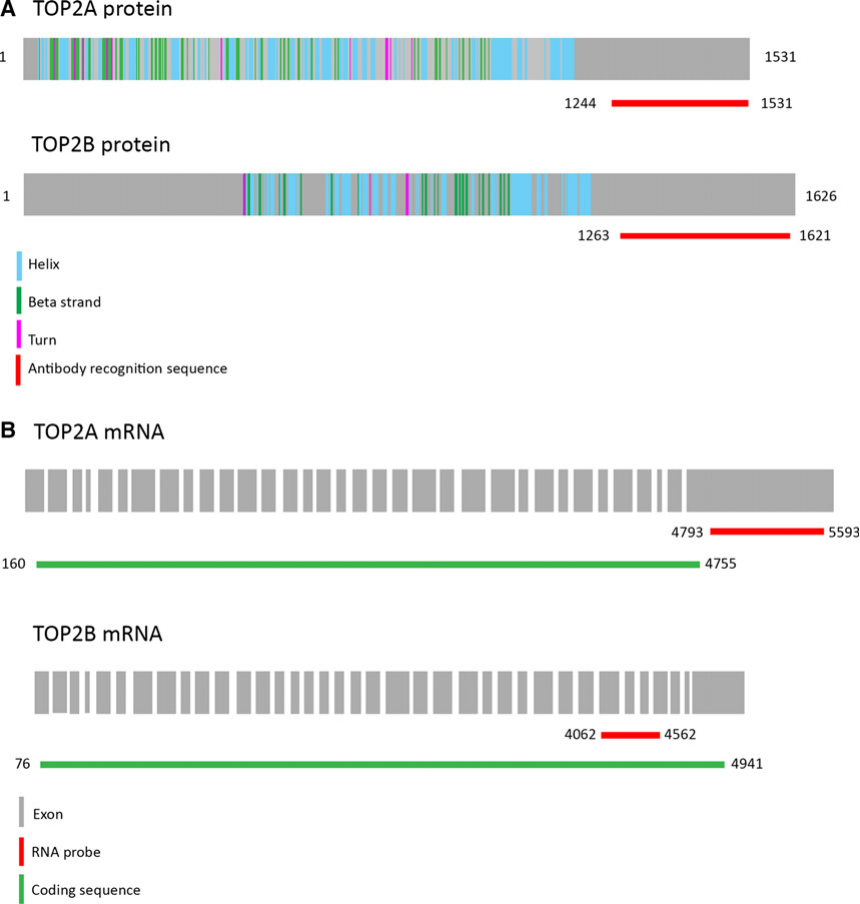
Schematic of TOP2A and B protein and mRNA showing the regions detected/amplified by IHC and ISH techniques. Polyclonal antibodies designed to recognise the C‐terminal domain of the TOP2A and TOP2B proteins were designed (A). Probes were manufactured to identify the 3′ UTR of TOP2A within exon 34 and a region spanning exons 30–34 near the end of the coding sequence for TOP2B (B). Control probes were designed for the same regions to identify any antisense transcripts or non‐specific staining.

**Figure 2:**
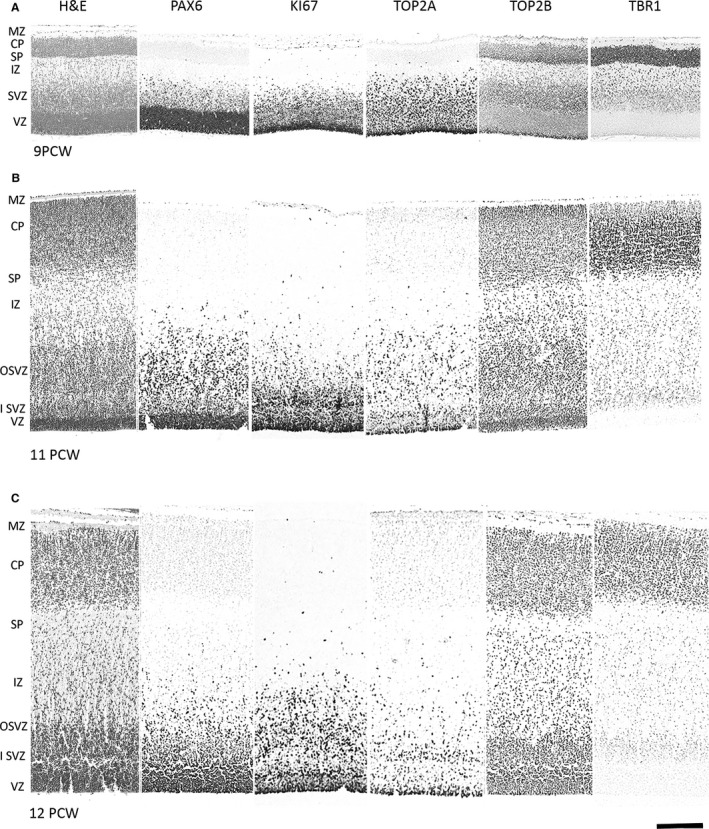
Immunoperoxidase staining for TOP2A and TOP2B in the human cerebral cortex. PAX6 transcription factor immunoreactivity identified the ventricular zone (VZ) and subventricular zone (SVZ) of the human cerebral cortex from 9 to 12 PCW. TOP2A immunoreactivity was confined to the proliferative ventricular and subventricular regions at 9, 11 and 12 PCW (A–C), and was similar in distribution to immunoreactivity for the cell division marker, KI67. Both were more prevalent in the inner than outer subventricular zone (ISVZ and OSVZ) that emerge by 11 PCW, unlike PAX6, which is unformly expressed throughout the SVZ. From 9 to 12 PCW, TOP2B immunoreactivity was present in the VZ and SVZ, as well as the intermediate zone (IZ), subplate (SP), post‐mitotic cortical plate (CP) and cell sparse marginal zone (MZ), all of which were also immunopositive for the transcription factor TBR1 (A–C). Scale bar: 250 μm.

TOP2A protein localisation was determined using antibodies that recognise the C‐terminal domain of the protein (Fig. 1A), residues 1244–1531 (Table 2). The transcription factor PAX6 is involved in cortico‐neurogenesis and expressed in the radial glial progenitor cells found in the VZ and SVZ of the developing human cerebral cortex (Bayatti et al. 2008a,b; Lui et al. 2011; Fig. 2). TOP2A immunoreactivity was detected in these proliferative regions at 9, 11 and 12 PCW (Fig. 2), although fewer cells were TOP2A‐positive than PAX6‐positive, suggesting that TOP2A protein is not found in all radial glial progenitor cells. Instead, the staining was more comparable to that of the marker of cell division KI67 (Scholzen & Gerdes, 2000; Fig. 2). TOP2A and KI67 both appeared very strongly at the apical surface of the VZ (Fig. 2), where cells are in the G2/M phase of division (Scholzen & Gerdes, 2000). Both markers also densely labelled cells in the inner subventricular zone (ISVZ; Fig. 2), where TBR2‐expressing intermediate progenitor cells are located (Bayatti et al. 2008a; Lui et al. 2011). A few cells of the intermediate zone (IZ) also stained positively for TOP2A at 9 PCW; however, as the IZ expands and becomes a more prominent feature of the cerebral cortex, fewer TOP2A‐immunopositive cells could be found (Fig. 2). The proliferative VZ and SVZ decreased in relative size as the cortex aged from 9 to 12 PCW due to the expansion of the post‐mitotic layers.

**Figure 3:**
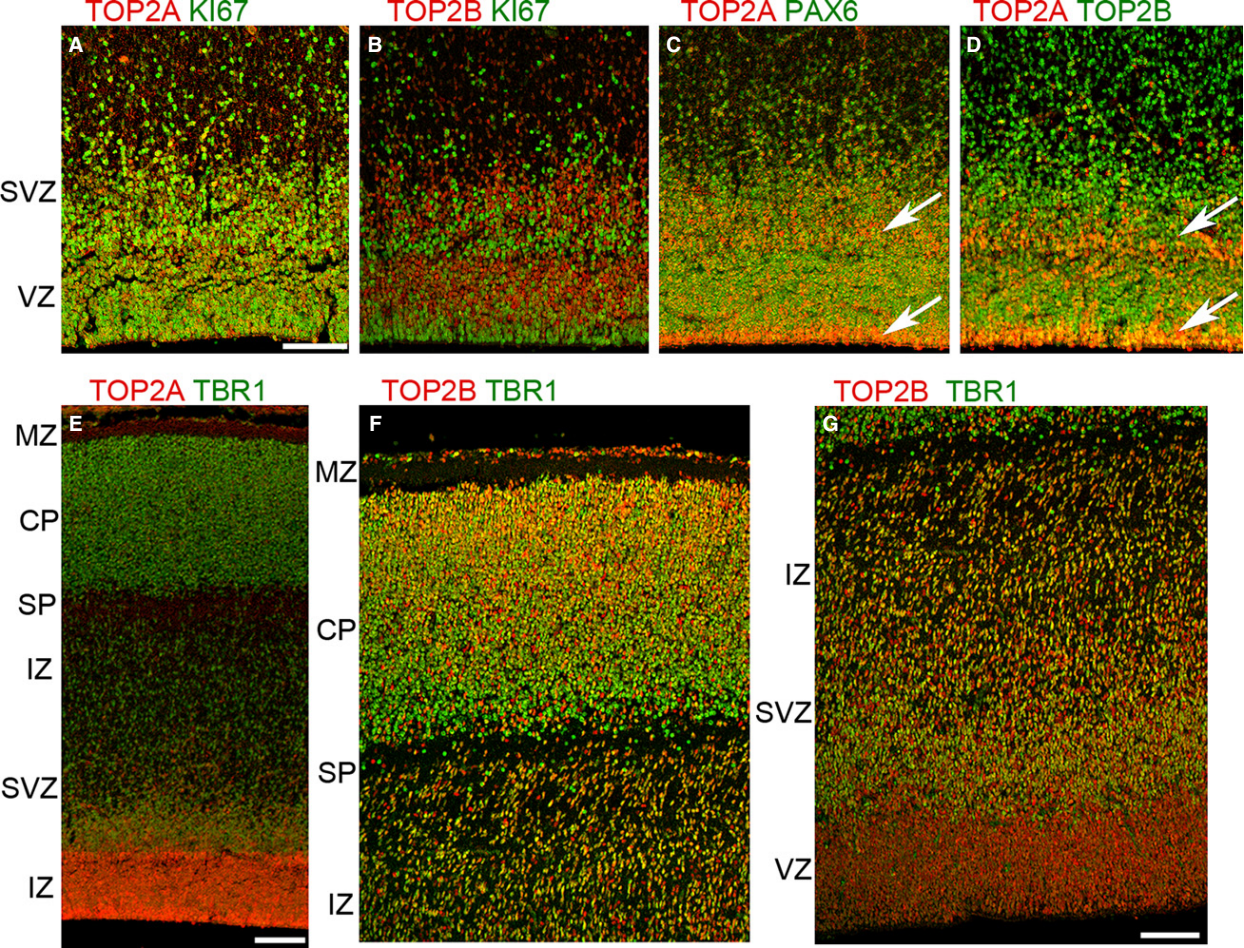
Immunofluorescent double‐labelling for TOP2A,TOP2B and other markers in the human cerebral cortex. (A–E) Taken from an 11 PCW foetus, (F, G) from 12 PCW. (A) Extensive co‐expression (pale green, yellow and orange cells) of TOP2A (red) and KI67 (green) immunofluorescence, whereas (B) demonstrates that there was very little co‐expression of TOP2B and KI67 as red and green cells predominate. (C) Not all PAX6‐expressing cells co‐expressed TOP2A, but those that did clustered close to the apical surface of the ventricular zone (VZ) and the border of the ventricular and subventricular zones (white arrows). Cells co‐expressing TOP2A and TOP2B also preferred these locations (D). (E) TOP2A and TBR1 show separation of expression although with a mingling of cells in the SVZ. On the other hand, (F) shows that TBR1 was co‐expressed with TOP2B in many cells in the cortical plate (CP), intermediate zone (IZ) and subventricular zone (SVZ; G), although cells expressing only TOP2A were observed throughout the CP and cells expressing TBR1 only were frequently seen in the deeper layers of the CP, marginal zone (MZ) and subplate (SP). Scale bars: 100 μm (in A for A–D, E, and in F for F and G).

To explore the extent of co‐localisation more precisely, double‐label immunofluorescence was attempted with a novel method that allowed the use of two primary antibodies from the same species and gave more sensitivity with paraffin sections than using conventional fluorescently conjugated secondary antibodies. It was possible to demonstrate that TOP2A was indeed co‐expressed with Ki‐67 (Fig. 3A), whereas not all PAX6‐positive radial glial cells were TOP2A‐positive (Fig. 3C), only those found at locations where KI67 was expressed, principally the apical surface of the VZ, and the ISVZ. As predicted, cells that express the post‐mitotic marker TBR1 did not express TOP2A (Fig. 3E). TBR1 expression was restricted to the CP, IZ and some cells of the SVZ. TBR1 was not expressed in the VZ and cells that expressed TBR1 in the SVZ did not co‐express TOP2A.

### TOP2B is expressed in both proliferative and post‐mitotic regions of the cortex

TOP2B protein localisation was determined using antibodies that recognise the C‐terminal domain of the protein (Fig. 1A), residues 1263–1621 (Table 1). TOP2B, in contrast to TOP2A, was observed throughout the cortex in layers occupied by both PAX6‐positive proliferative radial glia and TBR1‐positive post‐mitotic neurons (Englund et al. 2005; Bedogni et al. 2010; Fig. 2). At 9 PCW, TOP2B immunostaining was stronger in the SVZ and the CP than in the VZ, as was the case for TBR1 immunoreactivity (Fig. 2). At 9 PCW, the ventricular surface was immunopositive for TOP2B (Fig. 2) as it was for TOP2A, suggesting again that cells in the G2/M phase of cell division might express TOP2B as well as TOP2A. This is as expected as both TOP2B and TOP2A are expressed in undifferentiated cells (Thakurela et al. 2013). By 11 PCW (Fig. 2), staining was uniform throughout the VZ and was no longer stronger at the ventricular surface. Double‐labelling at 11 PCW showed that cells expressing KI67 did not express TOP2B (Fig. 3B). TOP2B was expressed in cells throughout the VZ and SVZ; however, these cells were not KI67‐positive. In comparison to this, some of the cells at the apical VZ and inner SVZ did express both TOP2A and TOP2B (Fig. 3D). This showed that not all of the cells expressing KI67 were immunopositive for TOP2A. Instead, there was a small proportion of TOP2A cells that may have ceased to express KI67 and have begun to co‐express TOP2A and TOP2B.

TBR1 expression was restricted to the CP, IZ and some cells of the SVZ. TBR1 was not expressed in the VZ and cells that express TBR1 in the SVZ did not co‐express TOP2A (Fig. 3E). Although devoid of TOP2A expression, the post‐mitotic CP was immunopositive for TOP2B from 9 to 12 PCW. Cells in this region have ceased division and have begun to differentiate into neurons. Fluorescent double‐labelling using TBR1 and TOP2B (Fig. 3F) confirmed that cells of the CP and IZ co‐expressed these markers. A closer look at the cells of the CP reveals that cells that co‐express both TBR1 and TOP2B were concentrated in the outer layers of the CP where younger cells have just finished radial migration from the proliferative zones (Rakic, 2009), whereas layers that contain the most mature neurons, the inner layer of the CP, as well as the SP and MZ, contained many cells that were only TBR1‐positive (Fig. 3F). Only a limited number of cells in this region were both TBR1 and TOP2B immunofluorescent. The majority of cells in the IZ expressed both TBR1 and TOP2B, and these may be radially migrating neuroblasts (Fig. 3F). A proportion of cells in the CP only expressed TOP2B, and these may be interneurons that do not express TBR1 as they are born in the GE (Hansen et al. 2013) but do express TOP2B (see below) and migrate tangentially to the CP (Hansen et al. 2013). There were a limited number of cells within the IZ and SVZ that expressed only TOP2B as is the case in the cells of the VZ (Fig. 3G). The presence of TOP2B and not TOP2A in the CP suggests a separate role for TOP2B in the post‐mitotic cortical cells of the developing cerebral cortex; however, TOP2B appears to be downregulated as the neurons mature.

### TOP2A is more prominent in the MGE than in the lateral ganglionic eminence (LGE)

**Figure 4:**
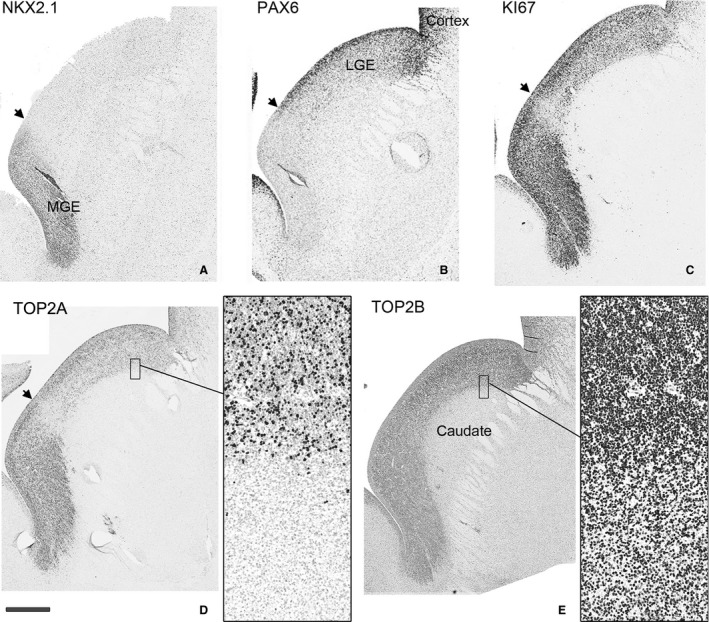
TOP2A and TOP2B immunoreactivity in the ganglionic eminences (GE). NKX2.1 immunopositivity (A) was predominantly observed in the medial ganglionic eminence (MGE), whilst PAX6 immunoreactivity (B) marked the lateral ganglionic eminence (LGE). The dividing cells in both the MGE and LGE were immunopositive for KI67 (C), TOP2A (D) and TOP2B (E). The medial part of the LGE showed a reduction in KI67, PAX6 and TOP2A immunoreactivity. TOP2B immunopositivity was also observed in the caudate and putamen (E), whereas TOP2A was absent from these post‐mitotic regions (D). (D and E) contain high‐magnification views of the transition between proliferative and post‐mitotic cell layers. Scale bar: 1 mm in low‐magnification images; 75 μm in high‐magnification insets.

**Figure 5:**
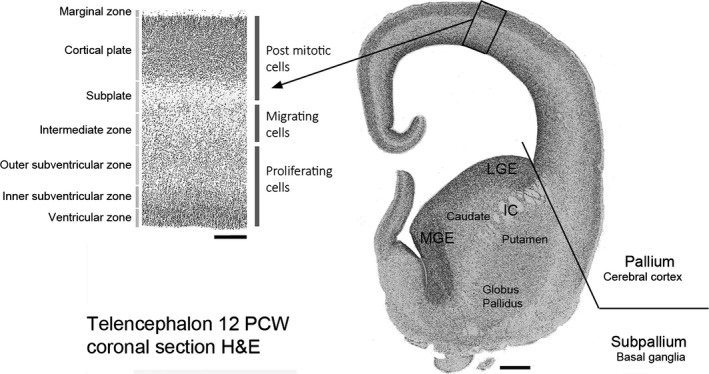
Haematoxylin and eosin (H&E) staining of a coronal section through the human telencephalon at 12 PCW. The ventricular zone (VZ) and subventricular zone (SVZ) of the cerebral cortex and the lateral and medial ganglionic eminences (LGE and MGE) contain proliferative cells. In the cortex, cells migrate from the VZ and SVZ, through the intermediate zone (IZ), to reach the post‐mitotic cortical plate (CP). The caudate, putamen and globus pallidus are regions containing post‐mitotic cells derived from the ganglionic eminences (GE). Scale bar: 1 mm in low‐magnification image; 200 μm in high‐magnification insert.

The cells of the GE can be split into distinct populations known as the LGE, MGE and caudal ganglionic eminence regions, all of which contain a high proportion of proliferative cells (Del Bigio, 2011). The location of the MGE was revealed by NKX2.1 immunoreactivity (Fig. 4A), whilst PAX6 immunoreactivity extended from the proliferative zones of the ventral pallium of the cortex into the LGE (Fig. 4B) but decreased in expression in a lateral to medial gradient. Both KI67 (Fig. 4C) and TOP2A (Fig. 4D) immunoreactivity were observed in the MGE and LGE; however, the immunoreactivity was less dense at the LGE : MGE boundary (arrowhead) compared with the density of cells shown by the H&E stain (Fig. 5). TOP2B immunoreactivity was observed throughout the MGE and LGE with no boundary apparent (Fig. 4E). TOP2A immunopositivity appeared to be stronger in the MGE (Fig. 4D) in comparison to TOP2B, which appeared to be stronger in the LGE (Fig. 4E). The emerging caudate nucleus, which contains post‐mitotic cells migrating away from the proliferative zones of the GE, showed high levels of TOP2B expression, but not TOP2A expression (Fig. 4D,E).

### *TOP2A* and *TOP2B* mRNA expressed at the apical surface of the VZ, and *TOP2B* expressed throughout the cortex

**Figure 6:**
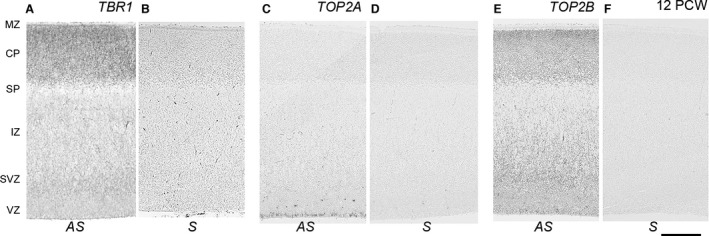
TOP2A and TOP2B mRNA expression in the cortex at 12 PCW. TBR1 mRNA was detected in the cortical plate (CP) of the cerebral cortex at 12 PCW, and was absent from the intermediate zone (IZ) and ventricular zone (VZ) (A). TOP2A mRNA was detected at the apical surface of the VZ (C) and in a small proportion of cells scattered throughout the VZ (C). TOP2B mRNA was detected throughout the cortex in both the proliferative VZ and subventricular zone (SVZ), the IZ and the post‐mitotic CP (E). Control experiments with sense transcripts to TBR1 (B), TOP2A (D) and TOP2B (F) showed no staining of the cortex at 12 PCW. Scale bar: 250 μm.

At 12 PCW, *TBR1* mRNA was located in the post‐mitotic cells of the CP (Fig. 6A). This corresponded with TBR1 protein localisation (Figs 2 and 3). *TOP2A* mRNA was restricted to the apical surface of the VZ as well as a small number of cells scattered throughout the VZ (Fig. 6C). This differed slightly from TOP2A protein localisation as the protein was found throughout the VZ and SVZ (Fig. 2). This suggests that *TOP2A* mRNA has a much shorter half‐life than the protein and that its expression is tightly controlled. *TOP2B* mRNA was detected throughout the cortex at 12 PCW in both proliferative and post‐mitotic regions (Fig. 6E). This matched the protein expression of TOP2B (Fig. 2).

## Discussion

It was found that TOP2A and TOP2B mRNA and protein are expressed in the VZ and SVZ of both the pallium and subpallium of the human telencephalon between 9 and 12 PCW. In addition, TOP2B was also expressed in post‐mitotic cells of the telencephalon, suggesting it may have a role in transcription in immature human neurons.

### Expression in the proliferative zones of the pallium

The precise localisation of TOP2A expression and the differences between protein and mRNA give clues to the nature of cell proliferation in human pallial proliferative zones. Far fewer cells expressed TOP2A than PAX6, the marker for radial glial progenitors, which is the major population of progenitor cells at this stage of primate cortical development (Lui et al. 2011; Betizeau et al. 2013). However, the majority of cells that express TOP2A also express KI67, the cell division marker that is expressed throughout all the phases of the cell cycle (Scholzen & Gerdes, 2000).

*TOP2A* mRNA was, however, more restricted in expression, suggesting that its half‐life is much less than the protein and is restricted to the G2/M phase, as it is seen predominantly in cells near to the apical surface of the VZ. During asymmetric division of apical radial glial cells, the nucleus undergoes interkinetic nuclear migration, shuttling between the basal boundary of the VZ and the apical (ventricular) surface in phase with the cell cycle, and is critical for self‐renewal of neural progenitor cells (Kosodo et al. 2004; Taverna & Huttner, 2010). TOP2A expression is known to increase throughout the S phase and peak during the G2/M phase, before being rapidly degraded during G1 (Heck et al. 1988; Woessner et al. 1991; Negri et al. 1992). Interestingly, although TOP2A and KI67 immunoreactiv‐ity were observed in the SVZ, and likely to be localised to dividing basal radial glia and intermediate progenitor cells (Lui et al. 2011; Betizeau et al. 2013), very little *TOP2A* mRNA expression was seen in this compartment. Perhaps cell division occurs less frequently in the SVZ or, alternatively, this reflects a difference in the dynamics of cell division once progenitors lose their apical process and cease to undergo interkinetic nuclear migration (Taverna & Huttner, 2010).

Cells of the VZ and SVZ showed either KI67 or TOP2B expression, suggesting that TOP2B is not involved in cell division. TOP2B also appears to be redundant during this process in human cell lines (Grue et al. 1998) and neurogenesis is normal in Top2b knockout mice (Yang et al. 2000). There are, however, cells that co‐express TOP2A and TOP2B in these proliferative regions suggesting that, as cells begin to differentiate, there is an overlap in type II topoisomerase expression during the transition from DNA replication to RNA transcription. *Top2b* is expressed by post‐mitotic neurons (Capranico et al. 1992; Tsutsui et al. 1993; Tiwari et al. 2012). Co‐expression of TOP2B with the post‐mitotic marker TBR1 in the human SVZ has been demonstrated. It is known that many cells in the human SVZ express several post‐mitotic neuronal markers, such as TBR1, MAP2 and CTIP2 (Bayatti et al. 2008a; Ip et al. 2011), and therefore the increased expression of TOP2B in the SVZ may reflect this.

### Expression in the subpallium

A complicated pattern of expression of TOP2A was observed across the subpallial compartments, but this most likely matches the extent to which cell division is taking place at each location, as it matches the pattern of KI67 expression. Reduced TOP2A expression at the medial boundary of the LGE coincides with a reduced density of dividing cells and PAX6‐positive radial glia. However, this does not prove a requirement for PAX6‐expressing radial glia in order to observe dividing cells as TOP2A expression is high in the MGE in the absence of PAX6. The MGE is the developmental source of pallidal projection neurons, striatal interneurons and about 50% of cortical inhibitory interneurons in the human (Marin et al. 2000; Xu et al. 2008; Hernandez‐Miranda et al. 2010; Hansen et al. 2013; Wang et al. 2013). Neural stem cells (VZ) and intermediate progenitors (SVZ) of the MGE instead express NKX2.1, and the SVZ of the MGE is expanded in size and complexity in primate compared with the rodent, presumably to provide increased numbers of interneurons to populate the relative larger cerebral cortex of primate species (Hansen et al. 2013). The high levels of TOP2A expression in this compartment may reflect this increased level of cortical interneuron progenitor activity.

### TOP2B expression in post‐mitotic neurons

As in rodents, TOP2B mRNA and protein is found in the post‐mitotic CP where no DNA synthesis is taking place, consistent with the suggestion that it regulates gene expression concerned with cell differentiation and survival (Thakurela et al. 2013). Cells that co‐express TOP2B and TBR1 are more apparent in the superficial layers of the CP, which are formed from the cells that have most recently migrated here from the proliferative regions, past the deep layer neurons (Rakic, 2009). This suggests that cells may lose TOP2B expression as they mature. At later stages of mouse development, both TOP2A and TOP2B expression in the brain decreases (Capranico et al. 1992).

Top2b knockdown is known to disrupt target finding of axons (Yang et al. 2000), and produce shorter neurite lengths and growth cone degeneration (Nur‐E‐Kamal et al. 2007). Top2b has been proposed as the main regulator of ganglion cell axon path‐finding during zebrafish retinal development (Nevin et al. 2011). Mouse retina‐specific Top2b knockout alters expression of a number of genes concerned with both cell survival and differentiation, and results in aberrant lamination of the retina as well as loss of synaptic connections in the inner and outer plexiform layers, and the degeneration of neurofilaments. Interestingly, neurexin genes 1 and 3, which code for cell adhesion molecules implicated in synapse formation and stabilisation, are significantly downregulated (Li et al. 2014). These genes have been identified as risk alleles for ASDs in human (Feng et al. 2006; Szatmari et al. 2007; Yan et al. 2008; Vaags et al. 2012; Dachtler et al. 2014). Inhibition or knockdown of Top2b in cultured murine neurons decreased the expression of a very similar set of genes greater than 67 kb in size many of which, in humans, are susceptibility genes for ASD and involved in synaptic adhesion and synapse formation, including NRXNs 1 and 3 (King et al. 2013). These NRXNS, along with other susceptibility genes such as CNTNAP2 and RELN included in the gene set, are known from microarray, qPCR, RNAseq and histological studies to be expressed in post‐mitotic compartments of the early human foetal cortex (Meyer et al. 2000; Abrahams et al. 2007; Ip et al. 2010; Kang et al. 2011; Konopka et al. 2012), suggesting ASD susceptibility genes may have roles in early developmental events such as cell migration and axon outgrowth, as well as late events such as synapse formation and stabilisation.
